# Electronic cigarettes versus nicotine-replacement therapy for smoking cessation: A systematic review and meta-analysis of randomized controlled trials

**DOI:** 10.18332/tid/154075

**Published:** 2022-10-20

**Authors:** Jing Li, Xu Hui, Jiani Fu, Muhammad Muneeb Ahmed, Liang Yao, Kehu Yang

**Affiliations:** 1 Health Technology Assessment Centre, School of Public Health, Lanzhou University, Lanzhou, People’s Republic of China; 2 Evidence-Based Medicine Center, School of Basic Medical Sciences, Lanzhou University, Lanzhou, People’s Republic of China; 3 Second Clinical Medical College, Lanzhou University, Lanzhou, People’s Republic of China; 4 Michael G. DeGroote School of Medicine, McMaster University, Hamilton, Canada; 5 Department of Health Research Methodology, McMaster University, Hamilton, Canada; 6 Key Laboratory of Evidence Based Medicine and Knowledge Translation of Gansu Province, Lanzhou, People’s Republic of China

**Keywords:** electronic cigarettes, nicotine-replacement therapy, continuous abstinence rate, 7-day point abstinence rate

## Abstract

**INTRODUCTION:**

Nicotine-replacement therapy (NRT) and electronic cigarettes (e-cigarettes) have been frequently used for smoking cessation. The aim of this review is to investigate the effectiveness and safety of e-cigarettes versus NRT for smoking cessation.

**METHODS:**

We searched PubMed, EMBASE, the Cochrane Library from inception to 10 October 2021. We included randomized controlled trials (RCTs) comparing e-cigarettes versus NRT for smoking cessation. Two authors independently screened titles, abstracts and full texts for eligibility. Paired authors extracted data, assessed risk of bias, and used GRADE (Grades of Recommendation, Assessment, Development, and Evaluation) to rate the certainty of evidence.

**RESULTS:**

The study included five RCTs with 1748 participants. The meta-analysis suggested the e-cigarettes versus NRT increased the ≥6 months continuous abstinence rate (RR=1.67; 95% CI: 1.21–2.28; 55 more per 1000 participants, low certainty), and 7-day point abstinence rate at ≥6 months follow-up (RR=1.43; 95% CI: 1.19–1.72; 84 more per 1000, low certainty). However, we found no evidence that e-cigarettes versus NRT increased 3–6 months continuous abstinence rate (RR=1.07; 95% CI: 0.73–1.57; 10 more per 1000, very low certainty) and <3 months continuous abstinence rate (RR=1.20; 95% CI: 0.90–1.60; 54 more per 1000, low certainty); similar results were found at <3 months follow-up (RR=1.19; 95% CI: 0.92–1.54; 55 more per 1000, very low certainty) and 3–6 months follow-up in 7-day point abstinence rate (RR=1.01; 95% CI: 0.70–1.44; 2 more per 1000, very low certainty). The adverse events were not significant between e-cigarettes and NRT other than throat irritation (RR=1.27; 95% CI: 1.13–1.42; 118 more per 1000, low certainty).

**CONCLUSIONS:**

E-cigarettes appeared to be superior to NRT in ≥6 months continuous abstinence rate and 7-day point abstinence rate. At short-term duration, we found no evidence that e-cigarettes compared to NRT increased the <6 months continuous abstinence rate and 7-day point abstinence rate.

## INTRODUCTION

The tobacco epidemic is one of the biggest public health threats the world has ever faced, an estimated 1.3 billion people worldwide use tobacco products, killing more than 8 million people a year around the world^1^. Quitting smoking is beneficial to health at any age; further, quitting smoking before the age of 40 years reduces the risk of death associated with continued smoking by about 90%^2,3^.

Nicotine-replacement therapy (NRT) including nicotine gum, patch, lozenges, sprays, and inhalers) and electronic cigarettes (e-cigarettes) have been used for decades in many countries for aiding smoking cessations^4-7^. Because of the poor compliance, the NRT are usually combined with behavioral support to quit smoking^8,9^. Compared with NRT, e-cigarettes received higher satisfaction ratings in smokers, due to the various flavors and popular shapes^10-12^. However, researchers found that e-cigarettes could increase the risk of short-term adverse events including mouth and throat irritation, dry cough, and nausea^13,14^, as well as the risk of long-term adverse events including asthma and COPD^12,14-16^, brain damage^17^, miscarriage, and abnormal brain development^18,19^.

Moreover, the effectiveness of e-cigarettes and NRT remains inconsistent; epidemiological studies showed that the smoking cessation in e-cigarettes users was 1.6–3.2 times higher than in NRT users^20-22^. While the randomized control trials (RCTs) evidence suggests conflicting results^23-25^. Although a current Cochrane review addressed e-cigarettes and NRT, the review did not conduct adequate subgroup analysis based on different duration of continuous abstinence rate, and did not include 7-day point abstinence rate in their analysis^26^. Therefore, we designed this comprehensive systematic review and meta-analysis to explore the different follow-up duration of smoking cessation rate between e-cigarettes and NRT.

## METHODS

### Search strategy

We performed a systematic review and meta-analysis of RCTs using a predefined protocol as per the Preferred Reporting Items for Systematic Reviews and Meta-analyses (PRISMA) recommendations^27-29^ (Supplementary file). Our study was registered in PROSPERO (CRD42020161815). We searched PubMed, Embase, and the Cochrane Library from inception to 10 October 2021. The computer-based searches combined search terms related to the intervention (e.g. electronic nicotine delivery systems, electronic cigarette, vaping, and e-cig) and control (e.g. nicotine-replacement therapy, NRT, nicotine patch, and nicotine gum) in humans, without language and publication year restrictions. The search strategy and specific terms used are listed in the Supplementary file. Two authors (XH and JL) independently screened titles and abstracts of all initially identified studies according to the selection criteria. Full-text articles of studies meeting the selection criteria were retrieved by two reviewers (JL and JNF), independently. Disagreements between evaluators were resolved by discussions with a third person.

### Selection criteria

We included RCTs assessing the smoking cessation of e-cigarettes and NRT in adults aged ≥18 years. We excluded non-randomized, observational studies, abstract, poster, letter, and other types of studies that did not undergo peer review. The patient important outcomes included continuous abstinence rate at <3 months, 3–6 months and ≥6 months, 7-day point prevalence of abstinence (the percentage of former smokers who are not smoking at a 7-day point in time^30^), and adverse events.

### Data extraction and quality assessment

Two authors (JL and JNF) extracted data, independently. In case of inconsistency, consensus was reached by discussions. A standardized predesigned data extraction form was used to obtain the relevant data from each study, including general information (e.g. country, study design, follow-up duration etc.), study participants (e.g. sample size, age, gender etc.), intervention description and outcomes of interest (e.g. continuous abstinence rate, 7-day point prevalence of abstinence, adverse events).

Potential sources of bias in RCTs were assessed using the Cochrane Collaboration’s risk of bias tool, which assesses the following 7 possible sources of bias: random sequence generation, allocation concealment, blinding of participants and personnel, blinding of outcome assessment, incomplete outcome data, selective reporting, and other bias^31^. For each domain, studies were classified as low, unclear, or high risk of bias^32^.

The GRADE (Grades of Recommendation, Assessment, Development, and Evaluation) system was used to evaluate the certainty of the underlying evidence^33^. The system classified certainty of evidence as high, moderate, low, or very low according to factors that might downgrade the certainty: risk of bias, inconsistency, imprecision, indirectness and publication bias^34,35^.

### Statistical analysis

Binary outcomes were presented as relative risk (RR) with 95% confidence interval (CI). The overall effect was pooled using random-effects model. Statistical heterogeneity among studies were measured by the I^2^ statistic and Q test^36^, with I^2^>50% and p<0.05 indicating moderate heterogeneity, and I^2^>75% and p<0.05 indicating high heterogeneity. We conducted a subgroup analysis according to short-term (<3 months), medium-term (3–6 months) and long-term follow-up (≥6 months) to explore the subgroup modifications. Unfortunately, given that the review involved fewer than 10 studies, we did not explore potential publication bias using a funnel plot and Egger intercept^37^. We used Review Manager 5.3 software to perform the meta-analysis.

## RESULTS

### Study selection

**Figure 1:**
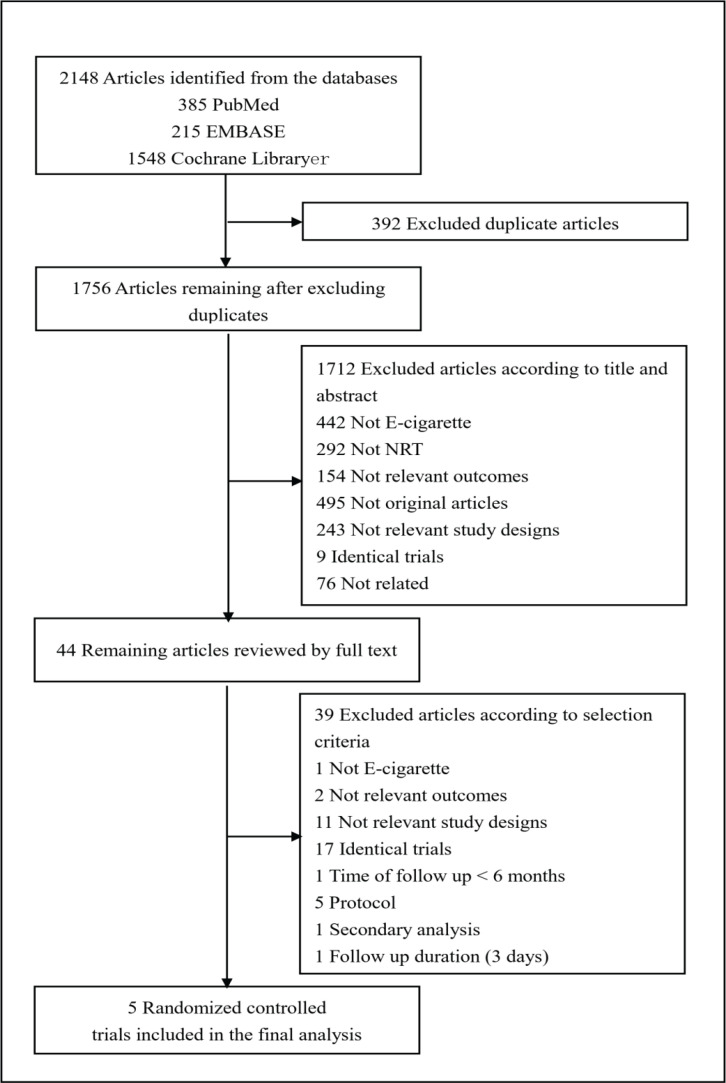
Identification of relevant randomized controlled trials

Figure 1 shows how we identified relevant randomized controlled trials. A total of 2148 articles were retrieved from the databases and relevant bibliographies. We excluded 392 duplicate articles and an additional 1712 articles that did not fulfill the selection criteria. After reviewing the full texts of the remaining 44 articles, we included 5 RCTs in the final analyses (Figure 1)^23-25,38,39^.

### Study characteristics

The characteristics of the included trials are presented in Table 1. The five eligible trials consist of a total of 1748 participants (872 in e-cigarettes group and 876 in NRT group), of whom, 67% were male, and the median age was 42 years. The NRT therapy was varied among studies: including nicotine patches in two studies^24,25^, nicotine gum^23^ in one study, and nicotine-replacement products in two studies^38,39^, one study was conducted in the US^25^, one each in Korea^23^, New Zealand^24^, UK^38^, and Australia^39^.

**Table 1:** Characteristics of the included studies

*Author Year*	*Country*	*Follow-up duration^#^ (months)*	*Participants*							*Products* *Intervention* *Control*	*Multi-center*	*Primary outcome*	*Secondary outcome*			*Definition of smoking cessation*
			*Total* *n*	*Intervention* *N*	*Control* *n*	*Mean age (years)*	*Male* *n (%)*	*Smoked (years) mean (SD)*	*Lost to follow-up n (%)*			*CAR Intervention Control E-cigarette use after treatment*	*Adverse event* *Intervention (%)*	*Adverse event* *Control (%)*	*7-day point abstinence rate* *Intervention* *Control*	
Bullen^24^ 2014^¶¶, ¶^	New Zealand	6	584	289	295	42.0	224 (38.36)	25.90 (13.10)	128 (21.9)	e-cigarettes nicotine patches	No	21/289 17/295 29%	Any adverse event (37) Serious adverse event*(7)	Any adverse event (33) Serious adverse event*(4)	61/289 46/295	A: self-reported abstinence over the whole follow-up period, allowing ≤5 cigarettes in total; B: proportion reporting no smoking of tobacco cigarettes, not a puff, in the past 7 days.
Lee^25^ 2018^¶^	USA	4.5	30	20	10	53.7	27 (90.00)	32.0 (15.6)	6 (20.0)	e-cigarettes nicotine patches	No	5/20 1/10 80%	Any adverse event (50) Serious adverse event (0); throat irritation (25); cough (40); nausea (25); headache (20)	Any adverse event (30) Serious adverse event (0); throat irritation (30); cough (10); nausea (10); headache (40)	NR	A: The definition of smoking cessation was verified by exhaled carbon monoxide ≤10 ppm; or self-report; B: NA.
Hajek^38^ 2019^¶¶, ¶^	UK	12	884	438	446	41.0	460 (52.04)	Age started smoking Median (IQR) 16 (14–18)	188 (21.3)	e-cigarettes nicotine-replacement products	Yes (3)	79/438 44/446 80%	Any adverse event (65) Serious adverse event** (13); throat irritation (65); cough (31); nausea (31)	Any adverse event (51) Serious adverse event** (13); throat irritation (51); cough (40); nausea (38)	146/438 98/446	A: self-report of smoking no more than five cigarettes from 2 weeks after the target quit date, validated biochemically by an expired carbon monoxide level of <8 ppm at 1 year follow-up and not contradicted by any previous self-report or validation result; B: NR.
Lee^23^ 2019	Korea	3	150	75	75	42.3	150 (100)	23.26 (7.60)	18 (12.0)	e-cigarettes nicotine gum	No	16/75 21/75 NR	Any adverse event (7) Serious adverse event (0); throat irritation (0); cough (4); nausea (1); headache (1)	Any adverse event (17) Serious adverse event (0); throat irritation (3); cough (4); nausea (11); headache (3)	17/75 22/75	NR
Bonevski^39^ 2021	Australia	6	100	50	50	40.9	67 (67.00)	NR	50 (50)	nicotine vaping products nicotine-replacement products	No	9/25 10/25 48%	Any adverse event (60) Serious adverse event (40)	Any adverse event (40) Serious adverse event (0)	7/25 9/25	A: self-report of smoking ≤5 cigarettes since the date. B: proportion reporting no smoking of tobacco cigarettes, not a puff, in the past 7 days

NR: not reported. NA: not applicable. CAR: continuous abstinence rate.

* Serious adverse event by convention includes death, life-threatening illness, admission to hospital or prolongation of hospital stay, persistent or significant disability or incapacity, congenital abnormality, medically important.

** Pneumonia, acute myocardial infarction, depression etc.

# We defined the follow-up duration as time after the intervention to the end of follow-up.

¶¶ Studies reported secondary outcomes of treatment adherence, relapse rate.

¶ Study reported acceptability or satisfaction of product. A: continuous abstinence rate. B: 7-day point abstinence rate.

The longest follow-up duration of ≥6 months was in three trials^24,38,39^, <6 months in two trials^23,25^. After treatment, 29–80% of the patients still used e-cigarettes.

### Risk of bias

**Figure 2:**
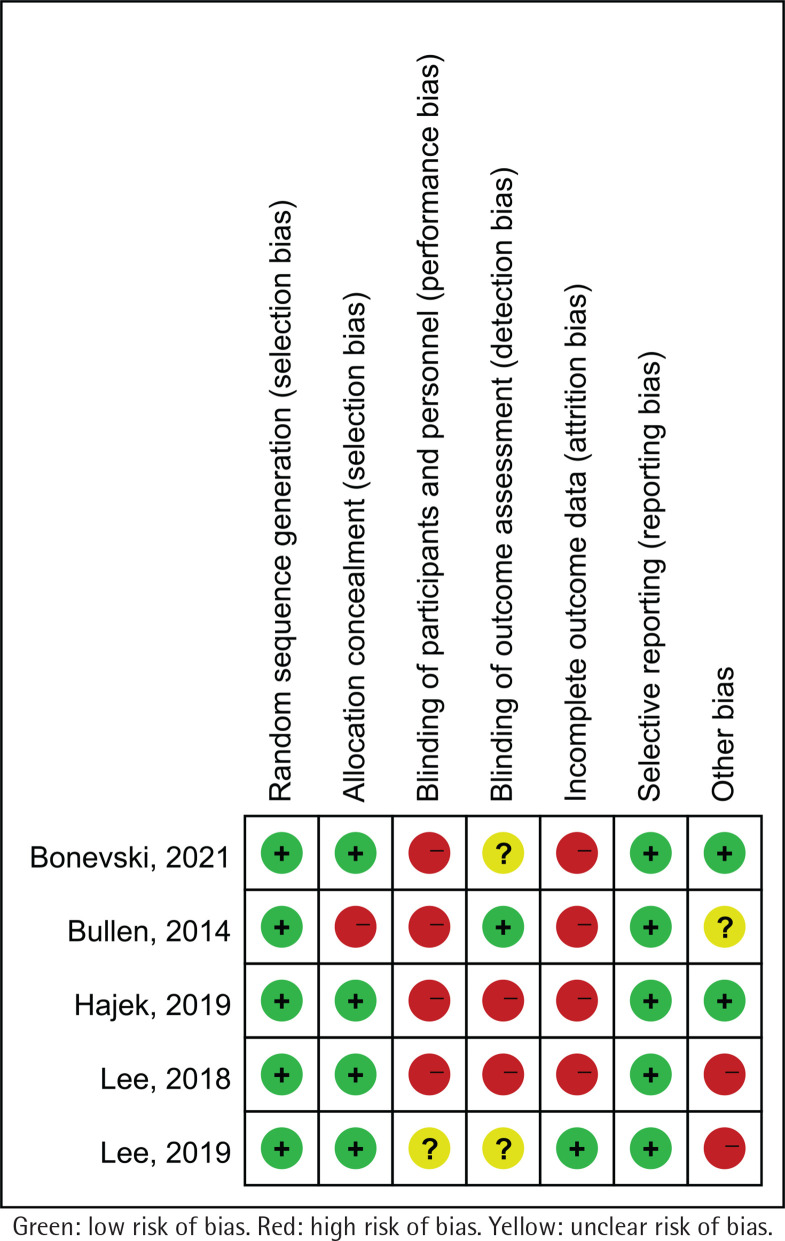
Summary of risk of bias for each trial

All the included studies had a low risk of bias in random sequence generation and selective reporting. Four trials^23,25,38,39^ were rated as low risk of bias and another one^24^ was rated as high risk in allocation concealment domain. Four trials^24,25,38,39^ were rated as high risk of bias and one^23^ was rated as unclear risk in performance bias. In the detection bias domain, two trials^25,38^ were rated as high risk of bias and one^24^ was rated as low risk. Four trials^24,25,38,39^ were rated as high risk of bias and one^23^ was rated as low risk in attrition bias domain (Figure 2 and Supplementary file Figure S1).

### Continuous abstinence rate

**Figure 3:**
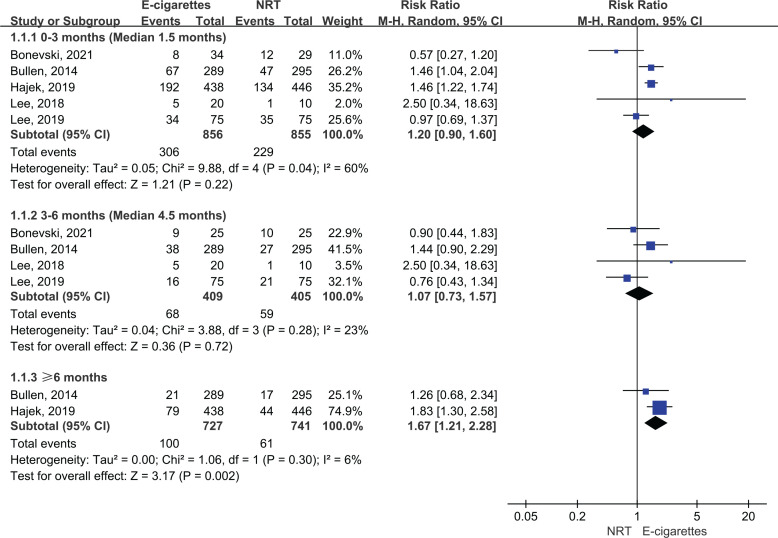
Subgroup analyses by follow-up duration in continuous abstinence rate in participants receiving e-cigarettes versus NRT

Figure 3 presents continuous abstinence rate outcomes. Both e-cigarettes and NRT could help increase smoking cessation rate at ≥6 months (14% vs 8%), 3–6 months (17% vs 15%), and at <3 months (36% vs 27%). The meta-analysis results suggest that e-cigarettes versus NRT was associated with higher continuous smoking cessation rate at ≥6 months (RR=1.67; 95% CI: 1.21–2.28; 55 more per 1000; low certainty). However, the continuous abstinence rate of e-cigarettes was not statistically significant at <3 months (RR=1.20; 95% CI: 0.90–1.60; 54 more per 1000, low certainty) and 3–6 months follow-up duration (RR=1.07; 95% CI: 0.73–1.57; 10 more per 1000, very low certainty) (Table 2 and Figure 3).

**Table 2:** Summary of findings of e-cigarettes (intervention) versus nicotine-replacement therapy (control) for smoking cessation

*Outcomes*	*Participants (RCTs)*	*Relative effect Change/1000 (95% CI)*	*Anticipated absolute effects*		Certainty of the evidence Grade**
			*Risk with NRT RR (95% CI)*	*Risk difference with e-cigarettes (per 1000)*	
Continuous abstinence rate <3 months (median: 1.5 months)	1711 (5)	54 (−27–161)	1.2 (0.9–1.6)	268	⨁⨁◯◯ Low^a,b^
Continuous abstinence rate 3–6 months (median: 4.5 months)	814 (4)	10 (−39–83)	1.07 (0.73–1.57)	146	⨁◯◯◯ Very Low^a,d^
Continuous abstinence rate ≥6 months	1468 (2)	55 (17–105)	1.67 (1.21–2.28)	82	⨁⨁◯◯ Low^a,b^
7-day point abstinence rate <3 months (median: 1.5 months)	1681 (4)	55 (−23–157)	1.19 (0.92–1.54)	292	⨁◯◯◯ Very Low^a,d^
7-day point abstinence rate 3–6 months (median: 4.5 months)	784 (3)	2 (−62–90)	1.01 (0.70–1.44)	205	⨁◯◯◯ Very Low^a,d^
7-day point abstinence rate ≥6 months	1468 (2)	84 (37–141)	1.43 (1.19–1.72)	196	⨁⨁◯◯ Low^a,b^
Adverse event: subjects with any adverse events	1684 (5)	82 (−12–197)	1.20 (0.97–1.48)	410	⨁◯◯◯ Very Low^a,c,d^
Serious adverse events	1684 (5)	24 (−2–106)	1.29 (0.73–2.28)	82	⨁◯◯◯ Very Low^a,c,d^
Adverse event: throat irritation	1050 (3)	118 (57–184)	1.27 (1.13–1.42)	437	⨁⨁◯◯ Low^a,b^
Adverse event: cough	774 (3)	−6 (−164–316)	0.98 (0.48–2.00)	316	⨁◯◯◯ Very Low^a,c,d^
Adverse event: nausea	1064 (3)	101 (−265–453)	0.70 (0.21–2.35)	335	⨁◯◯◯ Very Low^a,c,d^
Adverse event: headache	180 (2)	35 (−58–30)	0.50 (0.18–1.42)	71	⊕◯◯◯ Very Low^a,d^

a Downgraded by 1 level for serious risk of bias.

b Downgraded by 1 level for serious imprecision.

c Downgraded by 1 level for serious inconsistency.

d Downgraded by 2 level for very serious imprecision.

* The risk in the intervention group (and its 95% confidence interval) is based on the assumed risk in the comparison group and the relative effect of the intervention (and its 95% CI). RR: risk ratio.

** High certainty: we are very confident that the true effect lies close to that of the estimate of the effect. Moderate certainty: we are moderately confident in the effect estimate. The true effect is likely to be close to the estimate of the effect, but there is a possibility that it is substantially different. Low certainty: Our confidence in the effect estimate is limited. The true effect may be substantially different from the estimate of the effect. Very low certainty: We have very little confidence in the effect estimate: The true effect is likely to be substantially different from the estimate of effect. RCT: random controlled trail.

### 7-day point abstinence rate

**Figure 4:**
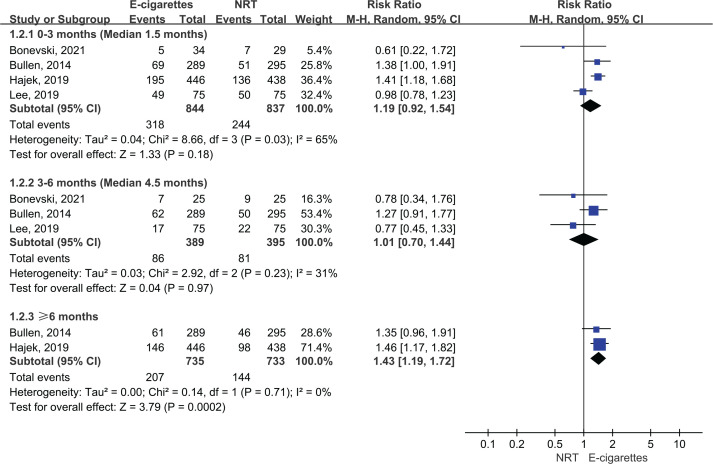
Subgroup analyses by follow-up duration in 7-day point abstinence rate in participants receiving e-cigarettes versus NRT

Five studies reported the 7-day point abstinence rate. The meta-analysis suggested that compared with NRT, e-cigarettes increased 7-day point abstinence rate at ≥6 months follow-up (28% vs 20%; RR=1.43; 95% CI: 1.19–1.72; 84 more per 1000, low certainty), but the benefits of e-cigarettes with regard to 7-day point abstinence rate were not statistically significant at 3–6 months follow-up (22% vs 21%; RR=1.01; 95% CI: 0.70–1.44; 2 more per 1000, very low certainty) and at <3 months (38% vs 29%; RR=1.19; 95% CI: 0.92–1.54; 55 more per 1000, very low certainty) (Figure 4).

### Adverse events

**Figure 5:**
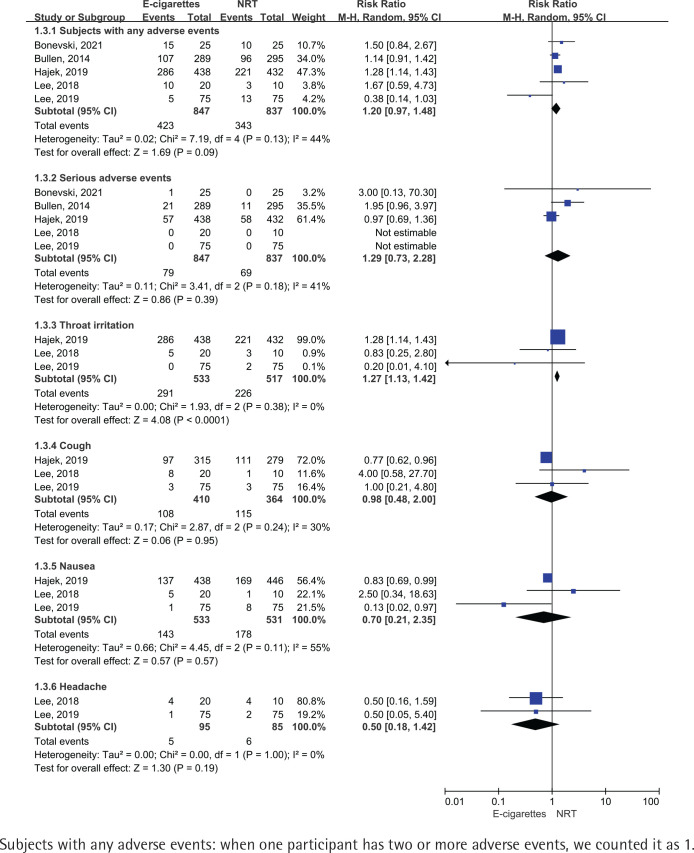
Adverse events in participants who received e-cigarettes versus NRT

We did not observe that the e-cigarettes group and NRT group differed in any adverse events (50% vs 41%; RR=1.20; 95% CI: 0.97–1.48; 82 more per 1000, very low certainty), serious adverse events such as pneumonia, acute myocardial infarction and asthmatic attack (9% vs 8%; RR=1.29; 95% CI: 0.73–2.28; 24 more per 1000, very low certainty), cough (26% vs 32%; RR=0.98; 95% CI: 0.48–2.00; 6 fewer per 1000, very low certainty), nausea (27% vs 34%; RR=0.70; 95% CI: 0.21–2.35; 101 fewer per 1000, very low certainty) and headache (5% vs 7%; RR=0.50; 95% CI: 0.18–1.42; 35 fewer per 1000, very low certainty), one exception for throat irritation (55% vs 44%; RR=1.27; 95% CI: 1.13–1.42; 118 more per 1000, low certainty) (Table 2 and Figure 5).

### Publication bias

Publication bias was not assessed since we included fewer than 10 studies.

## DISCUSSION

We found low certainty of evidence about e-cigarettes versus NRT was associated with a higher continuous abstinence rate and 7-day point abstinence rate at ≥6 months follow-up. However, the benefits of e-cigarettes in terms of continuous abstinence were not statistically significant at <6 months follow-up, as well as in 7-day point abstinence rate. We did not observe a significant difference in adverse events outcomes other than throat irritation.

We found interesting findings that both e-cigarettes and NRT increased continuous smoking cessation rate at ≥6 months (14% vs 8%), 3–6 months (17% vs 15%), and at <3 months (36% vs 27%), while along with the longer duration, the continuous smoking cessation rate decreased in both groups, but more significantly in the NRT group (27% to 8%); based on that, it might imply that the e-cigarettes may be more superior to NRT in long-term smoking cessation.

This review showed the very low certainty that e-cigarette was associated with higher risk of adverse events. The results may be unreliable due to potential dual use of e-cigarettes with conventional cigarettes. Research showed that dual use of e-cigarettes and combusted tobacco could lead to more adverse health effects than the use of either one alone; and perhaps it was the biggest risk of using e-cigarettes to treat tobacco dependence^40,41^.

Our findings are consistent with the results from the study of Grabovac et al.^42^, a systematic review including 3 RCTs^24,25,38^ involving 1498 participants, which concluded the e-cigarettes were more effective than NRT in smoking cessation (RR=1.69; 95% CI: 1.25–2.27). Our study included an additional two RCTs^23,39^ instead of addressing the surrogate outcomes and we focused on the patient important outcomes including continuous abstinence rate and 7-day point abstinence rate, the former has the advantage of being more stable over time and across studies than point prevalence rates. Advantages of the point prevalence rate are that it has the potential to be validated biochemically and it can also be viewed as being sensitive to the early effects of an intervention, such as attempts to quit that are not maintained^30^.

There also exists another review conducted by Hartmann et al.^26^, which included 4 RCTs (1924 participants) in e-cigarettes versus NRT (but one of which was an abstract involving 216 participants), the quit rates were higher in e-cigarettes than NRT (RR=1.53; 95% CI: 1.21–1.93). Their study did not address the subgroup analysis of different follow-up duration, for which we showed that there might exist modifications in the continuous abstinence rate for different durations in our study. Further, our study included one more eligible trial and included 7-day point abstinence rate as another important outcome in the analysis.

### Strengths and limitations

The strengths of our study include restricting inclusion of only RCTs, using the GRADE system to calculate absolute effects for each outcome and rate the certainty of evidence. Moreover, the subgroup analysis was conducted based on different follow-up durations to explore the impact of short-term, median-term and long-term follow-up duration on smoking cessation between e-cigarettes and NRT.

The present study also has limitations. First, the missing data ranged from 12% to 50% among the included studies, which can result in some bias. Second, the definitions of the continuous abstinence rate varied among studies, some were defined as no more than five cigarettes by self-report in the whole follow-up duration^24,38^, some were defined by biochemical indicators^25^, which might explain the partial heterogeneity of meta-analysis. Third, various dose and course of e-cigarette and NRT across studies may be a potential source of heterogeneity, the other source may be diversity of NRT regimens across studies, such as nicotine gum, patch, or inhalators. Finally, as no studies included pregnant women and considered the possible injury to vital fetal organs due to e-cigarettes^43^, our results do not apply to this special population.

### Implications

Policymakers should balance the benefits and harms of e-cigarettes and NRT before they make decisions. Even though our findings showed that e-cigarettes appeared to be more effective than NRT in long-term duration, the harms between e-cigarettes and NRT are still uncertain, hence further studies are required to address this issue.

Further, policymakers may also need to refer to cost-effectiveness analysis to get more information before deciding whether the e-cigarettes are more cost-effective than NRT. One RCT estimated the lifetime incremental cost-effectiveness ratio of e-cigarettes to be £65 per quality-adjusted life-year (QALY) (85% probability below £20000/QALY), which indicated e-cigarettes as a highly cost-effective cessation aid compared with NRT^44^. Additionally, regulation of e-cigarettes should be strengthened since data for 2019 from Canada, England, and the US, show regular use (≥20 days in the last 30 days) among those aged 16–17, 17–18 and 18–19 years to be 5.7%, 2.7% and 6.7%^45^, respectively, and multiple international cohort studies have consistently confirmed a strong correlation between e-cigarette use among adolescents and young adults and subsequent cigarette use^46,47^.

## CONCLUSIONS

Based on the limited low certainty evidence, e-cigarettes appear to be superior to NRT in continuous abstinence rate and 7-day point abstinence rate at long-term duration. At short-term duration, we found no evidence that e-cigarettes compared to NRT increased <6 months continuous abstinence rate and 7-day point abstinence rate. The paucity of reliable research decreases the confidence in the results.

## Data Availability

The data supporting this research can be found in the Supplementary file.
