# Supplementary material for: Electronic cigarettes versus nicotine-replacement therapy for smoking cessation: A systematic review and meta-analysis of randomized controlled trials
Source: Tob Induc Dis. 2022 Oct 20;20:90. doi: 10.18332/tid/154075 (PMC9582581; doi:10.18332/tid/154075)
Supplement: Supplementary file 1 [file TID-20-90-s1.pdf]

Supplementary file

Search Strategy

PubMed

#1 Search ((("Electronic Nicotine Delivery Systems"[Mesh]) OR "Nebulizers and Vaporizers"[Mesh]) OR "Drug Delivery Systems"[Mesh]) OR "Vaping"[Mesh]

#2 Search (((((((("Electronic Cigarette*"[Title/Abstract]) OR E-Cigarette*[Title/Abstract]) OR "E Cigarette*"[Title/Abstract]) OR E-Cig*[Title/Abstract]) OR "E Cig*"[Title/Abstract]) OR vape*[Title/Abstract]) OR vaping[Title/Abstract]) OR ecig*[Title/Abstract]) OR "electronic nicotine delivery system*"[Title/Abstract]

#3 #1 OR #2

#4 Search "Nicotine Chewing Gum"[Mesh]

#5 Search ((((((((("Nicotine patch*"[Title/Abstract]) OR "Nicotine replacement therapy"[Title/Abstract]) OR NRT[Title/Abstract]) OR "nicotine gum"[Title/Abstract]) OR "nicotine nasal spray"[Title/Abstract]) OR "nicotine lozenge*"[Title/Abstract]) OR "nicotine tablet*"[Title/Abstract]) OR "nicotine sublingual"[Title/Abstract]) OR "nicotine inhal*"[Title/Abstract]) OR "nicotine therap*"[Title/Abstract]

#6 #4 OR #5

#7 #3 AND #6

Cochrane Library

#1 MeSH descriptor: [Electronic Nicotine Delivery Systems] explode all trees

#2 MeSH descriptor: [Nebulizers and Vaporizers] explode all trees

#3 MeSH descriptor: [Drug Delivery Systems] explode all trees

#4 MeSH descriptor: [Vaping] explode all trees

#5 #1 OR #2 OR #3 OR #4

#6 ("Electronic Cigarette"):tiab,kw OR (E-Cigarette*) :ti,ab,kw OR ("E Cigarette*"):ti,ab,kw OR (E-Cig*):ti,ab,kw OR ("E Cig*"):ti,ab,kw

#7 (vape*):ti,ab,kw OR (vaping):ti,ab,kw OR (ecig):ti,ab,kw OR ("electronic nicotine delivery system*"):ti,ab,kw

#8 #6 OR #7

#9 #5 OR #8

#10 MeSH descriptor: [Nicotine Chewing Gum] explode all trees

#11 ("Nicotine patch*"):ti,ab,kw OR ("Nicotine replacement therapy"):ti,ab,kw OR (NRT):ti,ab,kw OR ("nicotine gum"):ti, ab ,kw OR ("nicotine nasal spray"):ti,ab,kw

#12 ("nicotine lozenge*"):ti,ab, kw OR ("nicotine tablet*"):ti,ab,kw OR ("nicotine sublingual"):ti,ab,kw OR ("nicotine inhal*"):ti,ab,kw OR ("nicotine replacement"):ti,ab,kw

#13 ("nicotine therap*"):ti,ab,kw

#14 #11 OR #12 OR #13

#15 #10 OR #14

#16 #9 AND #15

Embase

#1 'electronic cigarette'/exp OR 'nebulizer'/exp OR 'vaporizer'/exp OR 'drug delivery system'/exp OR 'vaping'/exp

#2 'electronic cigarette*':ab,ti OR 'e cigarette*':ab,ti OR 'e cig*':ab,ti OR vape*:ab,ti OR vaping:ab,ti OR ecig*:ab,ti OR 'electronic nicotine delivery system*':ab,ti

#3 #1 OR #2

#4 'nicotine chewing gum':ab,ti OR nrt:ab,ti OR 'nicotine patch*':ab,ti OR 'nicotine gum':ab,ti OR 'nicotine nasal spray':ab,ti OR 'nicotine lozenge*':ab,ti OR 'nicotine tablet*':ab,ti OR 'nicotine sublingual':ab,ti OR 'nicotine inhal*':ab,ti OR 'nicotine replacement':ab,ti OR 'nicotine therap*':ab,ti

#5 'nicotine patch'/exp OR 'nicotine replacement therapy'/exp OR 'nicotine gum'/exp OR 'nicotine nasal spray'/exp OR 'nicotine lozenge'/exp OR 'nicotine inhaler'/exp

#6 #4 OR #5

#7 #3 AND #6

Table S1. The details of intervention.

Author, year	Intervention	Control
Lee, 2019	nicotine 0.01 mg/mL e-cigarette, 12-weeks supplies	nicotine 2 mg/tablet nicotine gum, 12-week supplies.
Bullen, 2014	16 mg nicotine e-cigarettes, 12 weeks	nicotine patches (21 mg patch, one daily); 12 weeks
Hajek, 2019	18 mg/ml e-cigarettes, 3 months	nicotine-replacement products (patch, gum, lozenge, nasal spray, inhalator, mouth spray, mouth strip, and microtabs), Participants were informed about the range of nicotine-replacement products (patch, gum, lozenge, nasal spray, inhalator, mouth spray, mouth strip, and microtabs) and selected their preferred product. Use of combinations was encouraged, typically the patch and a faster-acting oral product. 3 months
Lee, 2018	e-cigarettes ad libitum for 3 weeks, the Gold (2.4%) e-cigarettes ad libitum for 2 weeks and the Study (0%) e-cigarettes ad libitum for the final week. The number of e-cigarettes issued corresponded to the reported baseline cigarettes smoked per day, calculated assuming one NJOY e-cigarette was equivalent to 10 cigarettes.	Those smoking an average of ten or more cigarettes per day were given the 21 mg/day patch for 3 weeks, the 14 mg/day patch for 1 week, the seven mg/day patch for 1 week, and the 0 mg/day patch for 1 week. Participants who reported smoking an average of less than 10 cigarettes per day at baseline were given the 14 mg/day patch for 3 weeks, the seven mg/day patch for 2 weeks, and the 0 mg/day patch for 1 week. The 0 mg/day patches were clear inert TegadermTM (3M, St. Paul, MN, USA) patches cut to the size and shape of the nicotine patch.
Bonevski, 2021	Participants randomized to group 2 received a NVP starter kit which included the device (Innokin Endura T22; 1.5ohm atomizer, 4.5-mL tank) and 4-week supply of nicotine e-liquid. The unflavored e-liquid provided contained vegetable glycol, purified water, and nicotine. The dosing schedule of e-liquid provided to participants was dependent on their nicotine dependence score as measured by the Heaviness of Smoking Index. Participants scoring in the high nicotine dependence category were assigned an initial 4-week e-liquid supply (total 8- × 10-mL bottles) consisting of 2- × 10-mL bottles of 18 mg e-liquid and 6- × 10-mL bottles of 12-mg e-liquid. This allotment allows for a 1-week supply of 18-mg e-liquid while participants in this group familiarized themselves with the use of the device. The second and third batches of e-liquid (which were mailed to participants following calls at weeks 3 and 7) consisted of 8- × 10-mL bottles of 12-mg e-liquid only. Participants scoring in the moderate- and low-dependence categories received 3- × 4-week supplies of 8- × 10-mL bottles of 12-mg e-liquid. Written information on the risks and benefits of vaping, and instructions on how to use NVPs and safe storage and handling was included. A 1-week supply of 21-mg nicotine patches was also provided for use while learning how to use the NVP effectively.	Participants randomized to this group received 12 weeks of NRT, with a 4-week supply of patches plus oral forms of NRT (gum, lozenges, and inhalators) in the discharge pack. Refills of NRT were provided after the initial 4-week period following phone contact with participants in weeks 3 and 7 (4-week supply mailed on each occasion). Thus, in total, participants received 3 × 4 weeks supply of NRT over the 12-week period. During the 3- and 7-week calls, they could specify their preferences for which types of NRT to be mailed to them.

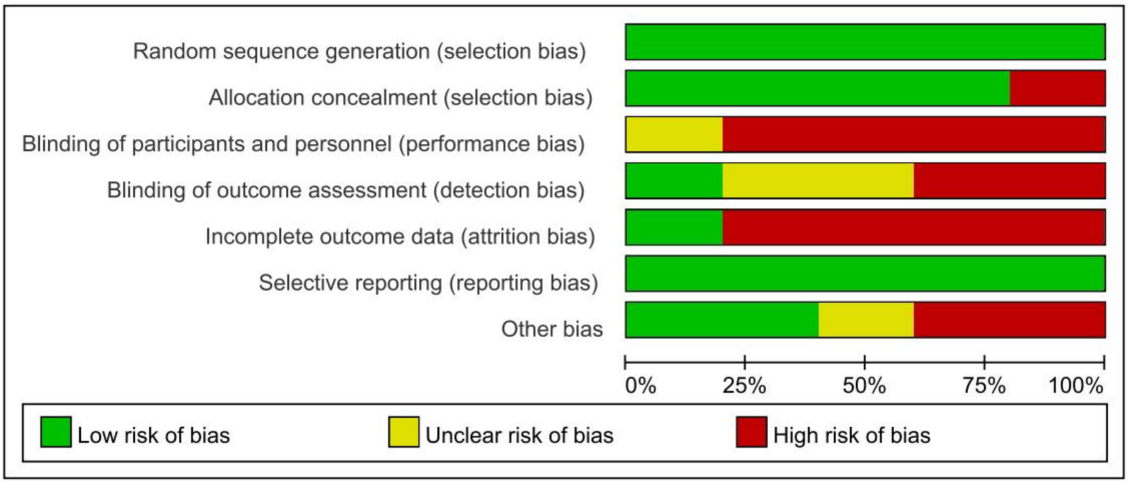

Fig S1. Risk of bias item presented as percentages across all included studies. Green=low risk of bias; red=high risk of bias; yellow=unclear risk of bias

PRISMA 2020 Checklist

Section and Topic	Item #	Checklist item	Location where item is reported
**TITLE**			1
Title	1	Identify the report as a systematic review.	
**ABSTRACT**			
Abstract	2	See the PRISMA 2020 for Abstracts checklist.	2
**INTRODUCTION**			
Rationale	3	Describe the rationale for the review in the context of existing knowledge.	3
Objectives	4	Provide an explicit statement of the objective(s) or question(s) the review addresses.	3
**METHODS**			
Eligibility criteria	5	Specify the inclusion and exclusion criteria for the review and how studies were grouped for the syntheses.	4
Information sources	6	Specify all databases, registers, websites, organisations, reference lists and other sources searched or consulted to identify studies. Specify the date when each source was last searched or consulted.	4
Search strategy	7	Present the full search strategies for all databases, registers and websites, including any filters and limits used.	4
Selection process	8	Specify the methods used to decide whether a study met the inclusion criteria of the review, including how many reviewers screened each record and each report retrieved, whether they worked independently, and if applicable, details of automation tools used in the process.	4
Data collection process	9	Specify the methods used to collect data from reports, including how many reviewers collected data from each report, whether they worked independently, any processes for obtaining or confirming data from study investigators, and if applicable, details of automation tools used in the process.	4
Data items	10a	List and define all outcomes for which data were sought. Specify whether all results that were compatible with each outcome domain in each study were sought (e.g. for all measures, time points, analyses), and if not, the methods used to decide which results to collect.	5
	10b	List and define all other variables for which data were sought (e.g. participant and intervention characteristics, funding sources). Describe any assumptions made about any missing or unclear information.	5
Study risk of bias assessment	11	Specify the methods used to assess risk of bias in the included studies, including details of the tool(s) used, how many reviewers assessed each study and whether they worked independently, and if applicable, details of automation tools used in the process.	5
Effect measures	12	Specify for each outcome the effect measure(s) (e.g. risk ratio, mean difference) used in the synthesis or presentation of results.	5
Synthesis methods	13a	Describe the processes used to decide which studies were eligible for each synthesis (e.g. tabulating the study intervention characteristics and comparing against the planned groups for each synthesis (item #5)).	5
	13b	Describe any methods required to prepare the data for presentation or synthesis, such as handling of missing summary statistics, or data conversions.	5
	13c	Describe any methods used to tabulate or visually display results of individual studies and syntheses.	5
	13d	Describe any methods used to synthesize results and provide a rationale for the choice(s). If meta-analysis was performed, describe the model(s), method(s) to identify the presence and extent of statistical heterogeneity, and software package(s) used.	5
	13e	Describe any methods used to explore possible causes of heterogeneity among study results (e.g. subgroup analysis, meta-regression).	5
	13f	Describe any sensitivity analyses conducted to assess robustness of the synthesized results.	5
Reporting bias assessment	14	Describe any methods used to assess risk of bias due to missing results in a synthesis (arising from reporting biases).	NA
Certainty assessment	15	Describe any methods used to assess certainty (or confidence) in the body of evidence for an outcome.	5

PRISMA 2020 Checklist

Section and Topic	Item #	Checklist item	Location where item is reported
**RESULTS**			
Study selection	16a	Describe the results of the search and selection process, from the number of records identified in the search to the number of studies included in the review, ideally using a flow diagram.	6
	16b	Cite studies that might appear to meet the inclusion criteria, but which were excluded, and explain why they were excluded.	6
Study characteristics	17	Cite each included study and present its characteristics.	9
Risk of bias in studies	18	Present assessments of risk of bias for each included study.	6
Results of individual studies	19	For all outcomes, present, for each study: (a) summary statistics for each group (where appropriate) and (b) an effect estimate and its precision (e.g. confidence/credible interval), ideally using structured tables or plots.	6
Results of syntheses	20a	For each synthesis, briefly summarise the characteristics and risk of bias among contributing studies.	6
	20b	Present results of all statistical syntheses conducted. If meta-analysis was done, present for each the summary estimate and its precision (e.g. confidence/credible interval) and measures of statistical heterogeneity. If comparing groups, describe the direction of the effect.	6
	20c	Present results of all investigations of possible causes of heterogeneity among study results.	6
	20d	Present results of all sensitivity analyses conducted to assess the robustness of the synthesized results.	NA
Reporting biases	21	Present assessments of risk of bias due to missing results (arising from reporting biases) for each synthesis assessed.	NA
Certainty of evidence	22	Present assessments of certainty (or confidence) in the body of evidence for each outcome assessed.	7
**DISCUSSION**			
Discussion	23a	Provide a general interpretation of the results in the context of other evidence.	8
	23b	Discuss any limitations of the evidence included in the review.	9
	23c	Discuss any limitations of the review processes used.	9
	23d	Discuss implications of the results for practice, policy, and future research.	10
**OTHER INFORMATION**			
Registration and protocol	24a	Provide registration information for the review, including register name and registration number, or state that the review was not registered.	4
	24b	Indicate where the review protocol can be accessed, or state that a protocol was not prepared.	4
	24c	Describe and explain any amendments to information provided at registration or in the protocol.	4
Support	25	Describe sources of financial or non-financial support for the review, and the role of the funders or sponsors in the review.	11
Competing interests	26	Declare any competing interests of review authors.	11
Availability of data, code and other materials	27	Report which of the following are publicly available and where they can be found: template data collection forms; data extracted from included studies; data used for all analyses; analytic code; any other materials used in the review.	11

From: Page MJ, McKenzie JE, Bossuyt PM, Boutron I, Hoffmann TC, Mulrow CD, et al. The PRISMA 2020 statement: an updated guideline for reporting systematic reviews. BMJ 2021;372:n71. doi: 10.1136/bmj.n71

For more information, visit: http://www.prisma-statement.org/

© 2022 Li J. et al.
